# Diagnose und Management der Osteoporose bei Diabetes mellitus (Update 2023): Gemeinsame Leitlinie der Österreichischen Gesellschaft für Knochen- und Mineralstoffwechsel und der Österreichischen Diabetes Gesellschaft

**DOI:** 10.1007/s00508-022-02118-8

**Published:** 2023-04-20

**Authors:** Christian Muschitz, Alexandra Kautzky-Willer, Yvonne Winhofer, Martina Rauner, Judith Haschka, Daniel Cejka, Robert Wakolbinger-Habel, Peter Pietschmann

**Affiliations:** 1 II. Medizinische Abteilung, Barmherzige Schwestern Krankenhaus Wien, Wien, Österreich; 2 Externe Lehre, Medizinische Universität Wien, Spitalgasse 23, 1090 Wien, Österreich; 3 Gender Medicine Unit, Klinische Abteilung für Endokrinologie und Stoffwechsel, Universitätsklinik für Innere Medizin III, Medizinische Universität Wien, Wien, Österreich; 4 Bone Lab Dresden, Medizinische Klinik und Poliklinik III, Medizinische Fakultät, Technische Universität Dresden, Dresden, Deutschland; 5 I. Medizinische Abteilung, Hanusch Krankenhaus, Wien, Österreich; 6 III. Medizinische Abteilung mit Nieren- und Hochdruckerkrankungen, Transplantationsmedizin und Rheumatologie, Ordensklinikum Linz Elisabethinen, Linz, Österreich; 7 Institut für physikalische Medizin und Rehabilitation, Klinik Donaustadt, Wien, Österreich; 8 Institut für Pathophysiologie & Allergieforschung, Zentrum für Pathophysiologie, Infektiologie und Immunologie, Medizinische Universität Wien, Wien, Österreich

**Keywords:** Diabetes, Osteoporose, Fraktur, Medikation, Diabetes-vermittelte Knochenerkrankung, Diabetes, Diabetes-related bone disease, Fracture, Medication, Osteoporosis

## Abstract

Diabetes mellitus und Osteoporose zählen zu den häufigsten chronischen Erkrankungen und kommen deshalb beide häufig in ein und demselben Individuum vor. Da die Prävalenz beider mit steigendem Alter zunimmt, wird in Anbetracht der Altersstruktur unserer Bevölkerung deren Häufigkeit zunehmen.

Patient:innen mit Diabetes haben ein erhöhtes Risiko für Fragilitätsfrakturen. Die Pathophysiologie ist unklar und vermutlich multifaktoriell.

Longitudinale Studien haben den Nachweis erbracht, dass das Fracture Risk Assessment Tool (FRAX) und die Knochendichte (BMD) mittels DXA (T-score) Messungen und einem eventuell vorhandenen Trabecular Bone Score (TBS) das individuelle Frakturrisiko vorhersagen können. Hierfür muss allerdings eine Adjustierung vorgenommen werden, um das Risiko nicht zu unterschätzen.

Es gibt derzeit aus osteologischer Sicht noch nicht den optimalen Ansatz, da es keine Studien mit rein diabetischen Patient:innen und Osteoporose gibt.

Patient:innen mit Diabetes mellitus und einem erhöhten Frakturrisiko sollten genauso wie Patient:innen ohne Diabetes und einem erhöhten Frakturrisiko behandelt werden.

Der Vitamin-D-Spiegel sollte auf jeden Fall immer optimiert werden und auf eine ausreichende Kalziumaufnahme (vorzugsweise durch die Nahrung) ist zu achten.

Bei der Wahl der antihyperglykämischen Therapie sollten Substanzen mit nachgewiesen negativem Effekt auf den Knochen weggelassen werden. Bei Vorliegen einer Fragilitätsfraktur ist auf jeden Fall – unabhängig von allen vorliegenden Befunden – eine langfristige spezifische osteologische Therapie indiziert.

Zur Prävention von Fragilitätsfrakturen sind antiresorptive Medikamente die erste Wahl, entsprechend den nationalen Erstattungskriterien auch anabole Medikamente. Das Therapiemonitoring soll im Einklang mit der nationalen Osteoporose Leitlinie erfolgen.

## Abstract (en)

Fragility fractures are increasingly recognized as a complication of both type 1 and type 2 diabetes, with fracture risk that increases with disease duration and poor glycemic control. The identification and management of fracture risk in these patients remains challenging. This manuscript explores the clinical characteristics of bone fragility in adults with diabetes and highlights recent studies that have evaluated areal bone mineral density (BMD), bone microstructure and material properties, biochemical markers, and fracture prediction algorithms (FRAX) in these patients. It further reviews the impact of diabetes drugs on bone tissue as well as the efficacy of osteoporosis treatments in this population. An algorithm for the identification and management of diabetic patients at increased fracture risk is proposed.

## Epidemiologie des Diabetes mellitus und osteoporotische Fragilitäts-Frakturen

Diabetes mellitus und Osteoporose zählen zu den häufigsten chronischen Erkrankungen und kommen deshalb beide häufig in ein und demselben Individuum vor, weshalb davon ausgegangen wird, dass sie in Zusammenhang stehen. Da die Prävalenz beider mit steigendem Alter zunimmt, wird in Anbetracht der Altersstruktur unserer Bevölkerung deren Häufigkeit zunehmen.

Rezente Metaanalysen mit rund 140.000 Patient:innen^1^ mit Typ 1 Diabetes (T1DM) zeigen ein gepooltes relatives Risiko (RR) für eine Fraktur von 3,16, für eine Hüftfraktur von 3,78 und für eine vertebrale Fraktur von 2,88. Das RR einer hüftgelenksnahen Fraktur bei Frauen mit T1DM im Vergleich zu Frauen ohne Diabetes beträgt 5,19 [1]. Das Frakturrisiko steigt mit zunehmendem Lebensalter an, speziell Hüftfrakturen treten bei T1DM etwa 10–15 Jahre früher auf [2].

Bei Typ 2 Diabetes (T2DM) weisen Populations-basierte Daten von rund 33.000 Patient:innen den T2DM als stärksten Prädiktor für niedrig-traumatische (= osteoporotische) Frakturen bei Männern (RR 2,38) und bei Frauen (RR 1,87) aus [3]. Mit einer durchschnittlichen Odds-Ratio (OR) für Frakturen von 1,5 ist der T2DM nur für rund 4 % aller osteoporotischen Frakturen ursächlich in Zusammenhang zu bringen. Dem gegenüber ist allerdings die global steigende Inzidenz von Patient:innen mit T2DM (rund 425 Mio. sowie rund 320 Mio. mit einer gestörten Glucose-Toleranz) gegenüber zu stellen. Zusätzlich zu diesem direkten Risiko kommen noch weitere klinische Risikofaktoren (clinical risk factors, CRF), die mit einem Diabetes einhergehen (z. B. multiple Stürze, Neuro- und Retinopathie, etc.) und das individuelle Frakturrisiko zusätzlich erhöhen (Tab. 1 und 2; [4]).

**Table 1:** 

Antidiabetogene Medikation	BMD	Frakturrisiko
*Metformin*	↔/↑	↓/↔
*Sulfonylharnstoffe*	KD	↓/(↑^a^)
*Thiazolidinedione*	↓↓/↔	↑↑
*Inkretin Mimetika*		
DPP4 Inhibitoren	↔	↓/↔
GLP1 Analoga	↑/↔	↔
*SGLT2 Inhibitoren*	↔	↔^b^
*Insulin*	↔/↓	↑

↑ Verbesserung/Erhöhung, ↓ Verminderung, ↔ unverändert

*KD* keine Daten, *DPP4* Dipeptidyl Peptidase‑4 Inhibitor, *GLP‑1* Glucagon-like peptide‑1 Analoga, *SGLT2* Natrium-Glucose Cotransporter 2, ^a^Hypoglykämie-assoziiertes erhöhtes Sturzrisiko

^b^Erhöhtes Frakturrisiko nur für Canagliflozin nachgewiesen

**Table 2:** 

Spezifisches Osteoporose Medikament	BMD (T2DM)	Frakturrisiko in Bezug auf T2DM
Alendronat	↑	KD/↔
Risedronat	↑	KD
Ibandronat	KD	KD
Zoledronat	KD	KD
Raloxifen	KD	↓/↔
Denosumab	KD	KD
Teriparatid	↑	↔

↑ Verbesserung/Erhöhung, ↓ Verminderung, ↔ unverändert

*KD* keine Daten

## Diabetes assoziierte Risikofaktoren für Frakturen

Diabetes per se ist ein klinischer Risikofaktor für ein erhöhtes Frakturrisiko (Tab. 3). Bei T2DM spielen das Alter und die Dauer des Diabetes eine wichtige Rolle. Sowohl bei Frauen als auch bei Männern > 40 Jahren ist T2DM ein unabhängiger Risikofaktor für sämtliche osteoporotische Frakturen (Hazard Ratio, HR 1,32). Das Alter beeinflusst das Risiko dahin gehend, dass jüngere Patient:innen ein höheres Risiko für Hüftfrakturen haben (HR Alter < 60 Jahre: 4,67; HR Alter 60–69 Jahre: 2,68; HR Alter 70–79 Jahre: 1,57; HR Alter > 80 Jahre: 1,42) [5]. Entscheidend ist außerdem die Dauer der Erkrankung. In den ersten fünf Jahren der Erkrankung kommt es zu keiner Erhöhung des relativen Risikos (ein protektiver Effekt vermehrter Fettmasse wird diskutiert), das Risiko folgt allerdings einem biphasischen Verlauf mit einem zweiten Gipfel jenseits von zehn Jahren Erkrankungsdauer (HR 1,47) [6].

**Table 3:** 

*Allgemeine Risikofaktoren*	FRAX CRF: Alter, Geschlecht, prävalente Fragilitätsfrakturen, Hüftfraktur der Mutter oder des Vaters, gegenwärtiges Rauchen, Alkohol (≥ 3 Einheiten/Tag), Glukokortikoide, Rheumatoide Arthritis, BMD Schenkelhals
*Krankheits-spezifische Risikofaktoren*	Dauer des Diabetes > 5 Jahre
	Antidiabetogene Medikamente: Insulin, TZDs, Canagliflozin, Sulfonylharnstoffe
	HbA1c > 7 %
	Mikrovaskuläre Komplikationen: periphere und autonome Neuropathie, Retinopathie, Nephropathie

*FRAX* Fracture Risk Assessment Tool der WHO, *CRF* clinical risk factors, *TZD* Thiazolidindione, *BMD* „bone mineral density“

Die glykämische Kontrolle ist wichtig für die Beurteilung des individuellen Frakturrisikos. Ein HbA1c > 7 % führt zu einem raschen Anstieg des Risikos mit einer erhöhten Mortalität nach Frakturen [7]. Zusätzlich hat eine schlechte glykämische Kontrolle einen negativen Einfluss auf die Mikroarchitektur des Knochens mit mikrovaskulären Komplikationen in diesem Organsystem [8].

## Diabetes, Niere & Knochen

Diabetes mellitus (DM) ist eine häufige Ursache für chronische Niereninsuffizienz (chronic kidney disease – CKD) auf Basis einer diabetischen Nephropathie (diabetic kidney disease – DKD). In Österreich ist DM (Typ 1 und 2) die häufigste Ursache für terminales Nierenversagen. Etwa 25 % aller Patient:innen, die in Österreich eine Nierenersatztherapie benötigen (Dialyse oder Transplantation), haben eine DKD als Grunderkrankung [1]. Sowohl DM als auch CKD erhöhen das Risiko für osteoporotische Frakturen. Im Vergleich zu nierengesunden Patient:innen mit DM haben Patient:innen mit DM und einer DKD ein etwa 1,4 bis 1,7-fach erhöhtes Risiko für osteoporotische Frakturen, einschließlich Hüftfrakturen [2, 3].

Als Basis jeder Behandlung von Patient:innen mit DKD wird eine Lebensstilmodifikation empfohlen [4]. Einige dieser Lebensstil-Empfehlungen wie sportliche Betätigung und Rauchstop sind auch aus osteologischer Sicht günstig. Darüber hinaus wird für die meisten Patient:innen mit einer DKD eine Therapie mittels ACE-Hemmern/Angiotensin-Rezeptor-Blockern (ACEi/ARB) in Kombination mit SGLT-2-Inhibitoren empfohlen [4].

In den letzten Jahren haben mehrere randomisierte prospektive Studien gezeigt, dass SGLT‑2 Inhibitoren sowohl in der Primärprävention (Vermeidung einer DKD) als auch in der Sekundärprävention (Behandlung einer bestehenden DKD) signifikante und klinisch relevante Vorteile bezüglich verlangsamter Progression der Niereninsuffizienz (verlangsamter GFR-Verlust), Reduktion der Albuminurie, Reduktion von akutem Nierenversagen und Reduktion des Auftretens einer terminalen (dialysepflichtigen) Niereninsuffizienz, bringen [5–11].

In einer SGLT-2-Inhibitor-Studie (Akronym: CANVAS) wurde eine erhöhte Rate osteoporotischer Frakturen unter Canagliflozin versus Placebo (hazard ratio 1,53) beobachtet [9]. Interessanter Weise kommt es unter SGLT-2-Inhibitor-Therapie zu einem (teilweise passageren und inter-individuell stark variierendem) Anstieg von Serum-Phosphat (vermutlich durch vermehrte renale Re-Absorption), Parathormon (PTH) und fibroblast growth-factor 23 (FGF-23) sowie einem korrespondierenden Abfall von Calcitriol (1,25-OH-D3) [12, 13]. Diese Auswirkungen einer SGLT-2-Inhibitor-Therapie auf den Mineralstoffwechsel könnten unter Umständen nachteilige Effekte auf die Knochengesundheit haben und auch eine Erklärung für die beobachtete Erhöhung der Frakturrate unter Canagliflozin sein.

In einer anderen prospektiven randomisierten Studie mit Canagliflozin wurde jedoch keine erhöhte Frakturrate gegenüber Plazebo gefunden [10], ebensowenig wie in allen weiteren Studien mit anderen SGLT-2-Inhibitoren (Empagliflozin [5, 6], Dapagliflozin [7, 8, 14], Ertugliflozin [15]). Auch in real-world-Analysen basierend auf Verschreibungsdaten und Gesundheitsdaten von Krankenversicherungen, Meta-Analysen und Analysen von Pharmakovigilanzmeldungen findet sich kein Hinweis für eine erhöhte Frakturrate unter einer Behandlung mit SGLT-2-Inhibitoren [16–19].

Zusammengefasst sind SGLT-2-Inhibitoren eine Standardtherapie zur Prävention und Behandlung einer DKD und haben keinen Einfluss auf das Frakturrisiko.

## Einfluss der Behandlung des Diabetes auf das Frakturrisiko

Das Verhältnis zwischen Diabetes und Knochenfragilität und die Identifizierung jener Patient:innen mit einem erhöhten Risiko für Frakturen wird zusätzlich durch die Eigenschaften antiglykämischer Medikamente auf das Skelett beeinflusst (Tab. 1). Obwohl es keine einzige prospektive Studie mit einem primären Studienziel in Bezug auf Therapie des Diabetes und Knochenfragilität gibt, zeigen Daten aus epidemiologischen und Beobachtungsstudien ein heterogenes Muster von teilweise positiven, aber auch negativen Effekten auf den Knochenstoffwechsel.

### Lebensstilfaktoren

Eine Veränderung des Lebensstils ist – nicht nur bei der Erkrankung Diabetes – eine der Säulen der nicht-medikamentösen Therapie. Grundsätzlich ist ein Gewichtsverlust, sofern keine Gegenmaßnahmen gesetzt werden, immer mit dem Verlust von Muskel- und Knochenmasse verbunden. Sarkopenie und sarkopene Adipositas sind Risikofaktoren für Stürze und Gebrechlichkeit, daher ist immer auf eine ausreichende alimentäre Zufuhr von Proteinen und progressives Widerstandstraining zu achten.

Körperliche Aktivität während einer gezielten Gewichtsabnahme verbessert die Lebensqualität und senkt gleichzeitig zirkulierende Sclerostin-Spiegel, unabhängig vom Alter der Patient:innen [20].

Andere nicht-pharmakologische Maßnahmen sind – wie bei vielen anderen Erkrankungen – die Vermeidung von Nikotin und übermäßigem Alkoholgenuss.

Ein Vitamin D Mangel ist sowohl beim T1DM als auch beim T2DM mit hoher Prävalenz vorhanden. Obwohl der direkte Beweis für die Wirksamkeit eines optimierten Vitamin D Spiegels bei Adipositas bzw. Diabetes und/oder Insulin-Resistenz noch nicht in Studien als primärer Endpunkt nachgewiesen wurde, ist doch davon auszugehen, dass Patient:innen mit Diabetes ähnlich wie nicht-diabetische Kollektive davon profitieren. Ein adäquater Vitamin D Spiegel und eine suffiziente Aufnahme von Calcium (vorzugsweise über die Nahrung) sind daher eine Grundvoraussetzung – auch im Hinblick auf die Prävention eines sekundären Hyperparathyreoidismus. Möglicherweise sind anfänglich höhere Einzeldosen von Cholecalciferol notwendig, um einen suffizienten Spiegel zu erreichen [21]. Eine Supplementation hat allerdings keinen Schutz vor Frakturen, Sturz oder klinisch relevante Effekte auf die Knochendichte gezeigt [22].

### Glykämische Kontrolle

Bei Patient:innen mit Diabetes besteht zusätzlich eine Fallneigung, welche wahrscheinlich zum erhöhten Frakturrisiko beiträgt. Die periphere Neuropathie, die Retinopathie mit Visusverschlechterung, vermehrte Stürze in der Anamnese, die Tendenz zu hypoglykämen Episoden, die Hypo- oder Hypertension bzw. die autonome Neuropathie sind hier beispielhaft zu nennen.

Eine eng eingestellte glykämische Kontrolle (HbA1c < 7 %) verringert das Frakturrisiko bei Diabetes, vor allem bei älteren Patient:innen. Allerdings ist sowohl die Hypoglykämie als auch die Hyperglykämie mit einem erhöhten Risiko für Fragilitätsfrakturen assoziiert, wahrscheinlich durch unterschiedliche Mechanismen [23]. Vor allem bei älteren Patient:innen mit Diabetes wird daher – um das Risiko für hypoglykäme Episoden zu vermeiden – eine weniger stringente Einstellung des Diabetes empfohlen, um das Sturzrisiko zu senken (EASD/ADA Guidelines) [24].

### Effekte antihyperglykämischer Therapie auf den Knochen

#### Metformin

In vitro Studien zeigen einen positiven Effekt von Metformin auf den Knochen durch Steigerung der Knochenmasse und Knochenstärke, einer Reduktion von AGEs (Advanced Glycolysation Endproducts) sowie einer Stimulation der Osteoblastogenese und einer verminderten Apoptose von Osteoblasten [25–27]. Präklinische und klinische Daten bestätigen einen neutralen bis positiven osteogenen Effekt in Bezug auf Frakturen und bei ketogener Ernährung, sodass diese Medikation in Bezug auf die Knochenqualität als sicher zu werten ist [28–32].

#### SGLT-2-Inhibitoren

Natrium-Glucose-Cotransporter 2 (SGLT2)-Inhibitoren gelten aufgrund ihrer zusätzlichen kardio-reno-protektiven Effekte als wichtige Substanzen in der Therapie des Typ 2 Diabetes. Sie hemmen im proximalen Tubulus die Reabsorption von Glucose und führen gleichzeitig zu einer vermehrten Reabsorption von Phosphat. Damit kann es potenziell zu einer Störung der Calcium-Phosphat-Homöostase kommen. Unter der Behandlung mit Dapagliflozin konnten leichte Anstiege von Magnesium, Phosphat und Parathormon gezeigt werden, jedoch ohne konsekutiven Effekt auf Serum-Calcium oder Vitamin D [33, 34]. Unter Dapagliflozin konnte zudem kein negativer Effekt auf den Knochenstoffwechsel oder die Knochendichte nachgewiesen werden 51. Sicherheitshinweise hinsichtlich verstärkter Knochenresorption und erhöhtem Frakturrisiko fanden sich lediglich bei der Behandlung mit Canagliflozin, wodurch eine FDA Warnung erfolgte. Daher sollte, wie auch in der täglichen Praxis üblich, einer Behandlung mit Dapagliflozin und Empagliflozin der Vorzug gegeben werden [35, 36]. Zusammenfassend fand sich in großen Meta-Analysen kein negativer Einfluss auf die Knochendichte sowie kein erhöhtes Frakturrisiko unter SGLT-2-Inhibitor Therapie [37].

#### Inkretinmimetika

##### GLP-1-Rezeptoragonisten

GLP-1-Rezeptoragonisten werden je nach Wirkdauer einmal täglich oder einmal wöchentlich verabreicht. Neben ihrer ausgeprägten blutzuckersenkenden Potenz, zeigen sie zudem positive Effekte auf das Körpergewicht und einen kardiovaskulären Benefit, weswegen jene mit nachgewiesenem kardiovaskulärem Benefit vor allem bei Patient:innen mit etablierten kardiovaskulären Erkrankungen oder einem hohen Risiko hierfür eingesetzt werden sollten.

Ein positiver Effekt der GLP-1R-Agonisten konnte durch eine gesteigerte Proliferation mesenchymaler Stammzellen, einer Adipozytendifferenzierung und einer verminderten Sclerostin Exprimierung gezeigt werden [38, 39]. Klinische Studien zeigten einen neutralen Effekt auf die Knochendichte unter GLP-1R-Agonisten Therapie [40]. Die aktuelle Datenlage zeigt kein erhöhtes Frakturrisiko durch den Einsatz von GLP-.1-RA; im Gegenteil, es gibt zunehmend Evidenz auf einen anti-osteoporotischen Effekt durch Verbesserung der Knochendichte und Qualität sowie Hemmung der Knochenresorption [41–43].

##### DPP-IV-Hemmer

Dipeptidyl-Peptidase-4-Inhibitoren (DPP4-Inhibitoren) zeigen bei T2DM ein mehrheitlich neutrales Sicherheitsprofil für das Skelettsystem. Während präklinische Studien eine Reduktion der Knochenresorption und eine Zunahme von trabekulärem und kortikalem Knochenvolumen unter DPP4-Inhibitor Therapie zeigten [25, 26, 38–40, 44, 45], ist in klinischen Studien eine neutraler bis positiver Effekt hinsichtlich Frakturprävention unter DPP-4-Inhibitor Therapie beschrieben, wenngleich keine dieser Untersuchungen die Frakturprävention als primären Endpunkt hatten und die Fallzahl an Frakturen gering war [46–48]. Zusammenfassend zeigt die aktuelle Datenlage einen neutralen bis positiven Effekt auf die Reduktion des Frakturrisikos.

##### Sulfonylharnstoffe

Unter Behandlung mit Sulfonylharnstoffen wurde eine gesteigerte Osteoblastenproliferation und Differenzierung in vitro gezeigt [25, 28]. Epidemiologische Daten wiederum zeigen unterschiedliche Ergebnisse in Bezug auf das Organsystem Knochen, mit einem neutralen bis positiven Effekt auf das Frakturrisiko. Daten zu Veränderungen der Knochendichte liegen nicht vor. Eine Auswertung von Hypoglykämie-assoziierten Events fand einen Zusammenhang mit einem erhöhten Risiko für Sturz-assoziierte Frakturen [46]. Zusammenfassend kann daher das Frakturrisiko als reduziert, mit Ausnahme des indirekt erhöhten Sturzrisikos bei Hypoglykämie, zusammengefasst werden [28, 31, 47].

##### Thiazolidinedione

Thiazolidinedione interagieren mit dem peroxisome proliferator-activated receptor (PPAR)γ, was zu einer Dysbalance zugunsten der Differenzierung von Adipozyten und zu Lasten der Differenzierung von Osteoblasten führt. Darüber hinaus kommt es zu einer gesteigerten Osteoklastogenese [25, 26, 48–50]. Eine große epidemiologische Auswertung von über 32000 Patient:innen mit Diabetes Mellitus Typ 2 bestätigte das erhöhte Frakturrisiko unter Behandlung mit Thiazolidinedionen, insbesondere für periphere Frakturen und bei Frauen unter 64 Jahren [28, 51]. Eine rezente Metaanalyse bestätigt ebenfalls den negativen Einfluss sowohl von Rosiglitazon, als auch von Pioglitazon auf den Knochen [32]. Es wird daher empfohlen, bei allen Patient:innen mit erhöhtem Risikoprofil für Fragilitätsfrakturen, insbesondere bei postmenopausalen Frauen, primär nicht einzusetzen [31, 52, 53].

##### Insulin

In präklinischen Studien konnte ein anaboler Effekt von Insulin auf den Knochen nachgewiesen werden. Im Kontrast dazu findet sich jedoch in klinischen Studien ein erhöhtes Frakturrisiko bei Patient:innen mit T2DM unter Insulin Therapie, insbesondere für nicht-vertebrale Frakturen [54–57]. Eine rezente epidemiologische Analyse in Österreich konnte das deutlich erhöhte Risiko für Hüftfrakturen bei Patient:innen unter Insulintherapie bestätigen [58]. Ursächlich muss die längere Krankheitsdauer und/oder eine schlechtere glykämische Kontrolle in Zusammenhang mit allen Sekundärkomplikationen der Erkrankung (Retinopathie, Neuropathie, Nephropathie, etc.) berücksichtig werden. Patient:innen unter einer Insulin-Therapie haben zudem indirekt durch Hypoglykämie-induzierte Stürze ein erhöhtes Risiko [59]. Wenig Evidenz liegt zu Veränderung der Knochenmineraldichte vor, wobei hier ein neutraler bis negativer Effekt insbesondere bei Männern zu beobachten war [60]. Zusammenfassend muss daher die Insulin Therapie als Risikofaktor für ein erhöhtes Frakturrisiko gesehen werden [28].

## Diagnostik

### DXA-Knochendichtemessung

Die Knochendichtemessung mittels DXA (dual energy x‑ray absorptiometry) ist nach wie vor der Goldstandard. Die Definition einer Osteoporose von einem T‑score ≤ −2,5 basiert auf einer Definition der WHO aus dem Jahr 1994 und definiert die Erkrankung, jedoch nicht die individuelle Interventionsschwelle [61].

Die Mehrzahl der Studien bei Patient:innen mit T1DM zeigen, dass die BMD bei dieser Patient:innenpopulation deutlich vermindert ist [62]. Aufgrund der meist vorherrschenden Adipositas als Risikofaktor bei T2DM wäre grundsätzlich davon auszugehen, dass ein hoher Body Mass Index (BMI) und eine hohe BMD positiv miteinander korrelieren. Daher haben Patient:innen mit einem T2DM in der Regel eine 5–10 % höhere BMD im Vergleich zur nicht-diabetischen gesunden Population. Die höhere BMD ist vor allem beim jüngeren männlichen Geschlecht vorherrschend – interessanter Weise auch bei höheren HbA1c Werten. Die höhere BMD ist vor allem am Gewichts-tragenden Knochen zu sehen, jedoch nicht am Radius.

**Figure 1:**
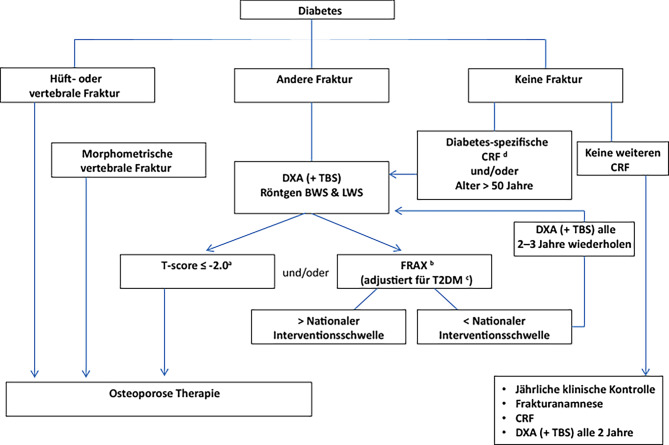

Die relativ höhere BMD bei T2DM schützt die Patient:innen jedoch nicht vor Frakturen. Die Mehrzahl der Patient:innen mit Frakturen haben einen T‑score im osteopenen Bereich, also einem T‑score > −2,5 [63]. Bei Frauen mit T2DM ist das individuelle Frakturrisiko im Gegensatz zu Frauen ohne Diabetes in etwa 0,5 T-scores als Korrekturfaktor tiefer als der tatsächliche Messwert anzusetzen (Abb. 1). Obwohl zahlreiche Studien in dieser Patient:innenpopulation bestätigen, dass die DXA-Messung systematisch das Frakturrisiko unterschätzt, werden unter Berücksichtigung dieses Korrekturfaktors vor allem ältere Patient:innen adäquat stratifiziert [64].

Manche Studien bestätigen einen schnelleren Verlust an BMD auch an Gewichts-tragenden Knochen (z. B. Hüfte) unter T2DM als möglichen Grund für die erhöhte Frakturrate [65].

Mit dem Trabecular Bone Score (TBS) steht eine Methode zur Verfügung, um aus einer zweidimensionalen DXA-Untersuchung Informationen über die Knochenmikrostruktur der Lendenwirbelsäule zu generieren. Diese einheitslose Zahl spiegelt anhand der Analyse von Grauwert-Variogrammen der radiologischen Messung der Lendenwirbelkörper L1–L4 mit einer hohen Korrelation die trabekuläre Mikroarchitektur bei Osteoporose unabhängig von der BMD wider [66]. Der TBS-Software kann direkt im Rahmen der Messung mit, oder auch retrospektiv den Score berechnen, wodurch sich keine zusätzliche Strahlenbelastung für den Patient:innen ergibt. Im Gegensatz zur DXA-Methode ist der TBS bei Patient:innen mit T2DM tiefer als in einer nicht-diabetischen Population. In einer großen Populations-basierten Untersuchung wurden Patient:innen mit einem TBS von < 1230 als Risikopatient:innen für Osteoporose-assoziierte Frakturen eingestuft, bei einem TBS von 1230–1310 ein mittleres Risiko. Bei T2DM fanden sich in Studien TBS Werte zwischen 1100 und 1200 [67].

Der TBS ist bei Patient:innen mit guter glykämischer Kontrolle höher und tiefer bei einem schlecht eingestellten T2DM. Der TBS ist somit ein unabhängiger Prädiktor für das Frakturrisiko bei Diabetes (HR 1,27) bzw. auch ohne Diabetes (HR 1,31) [68].

Alternative Methoden wie etwa der Ultraschall am Calcaneus oder am Radius zeigen inkonklusive Ergebnisse bei T2DM [69].

## Mikroarchitektur und Knochenqualität

Die BMD alleine – vor allem beim T2DM – erklärt nicht die erhöhte skeletale Fragilität. Sowohl in MRT-Untersuchungen als auch mittels HR-pQCT (high resolution peripheral quantitative computed tomography) am Radius (nicht Gewichts-tragender Knochen) und an der Tibia (Gewichts-tragender Knochen) zeigt sich beim T2DM eine verschlechterte Mikroarchitektur. Die Trabekel beim T2DM sind im Vergleich zum nicht-diabetischen Patient:innen eher hypertrophiert. Im trabekulären Netzwerk finden sich allerdings auch größere Löcher, zusätzlich ist die kortikale Porosität (bis zu 16 %) gegenüber Patient:innen ohne T2DM erhöht. Die strukturelle Alteration mit hoher Heterogenität ist an der endokortikalen Übergangszone besonders ausgeprägt („Trabekularisierung der Kortikalis“). Zusätzlich gibt es einen geschlechtsspezifischen Unterschied mit schlechteren Werten beim weiblichen Geschlecht [70, 71].

**Figure 2:**
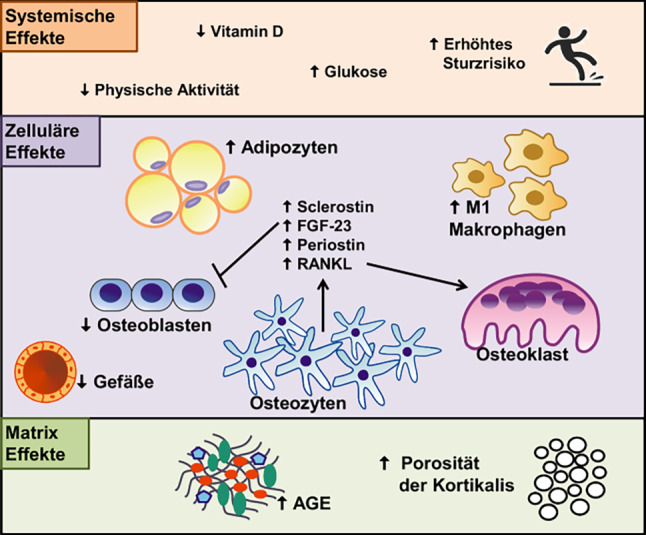

Aufgrund dieser strukturellen Defizite bei T2DM ist die Knochenfestigkeit sowie die Steifigkeit und die Elastizität des Knochens in virtuellen FEA (finite element analysis) Untersuchungen vermindert. Microindentations-Untersuchungen zeigen zusätzlich strukturelle Einschränkungen als Ausdruck veränderter Kollagenverlinkungen in der Knochenmatrix aufgrund vermehrter AGEs (advanced gylcation endproducts). Diese Verlinkungen führen dazu, dass der Knochen an Elastizität und Flexibilität verliert. In Summe haben diese Untersuchungsergebnisse den Begriff der „Diabetoporose“ geprägt ([72]; Abb. 2).

## Knochenstoffwechsel: Histomorphometrie und Serum Marker

Der Goldstandard zur Untersuchung des lokalen Knochenstoffwechsels ist die Histomorphometrie aus bioptischen Proben. Zur Abschätzung der tatsächlichen Aktivität ist einer der wichtigen Parameter die Formationsrate bezogen auf eine Referenzoberfläche der Biopsie (BFR/BS, bone formation rate/bone surface). Bei diabetischen Patient:innen ist dieser Parameter im trabekulären, endokortikalen und intrakortikalen Bereich um bis zu 70–80 % vermindert [73].

In der Mehrzahl der Studien wurde bei Patient:innen mit T2DM eine verminderte Aktivität von serologischen Formationsmarkern (procollagen type I N‑terminal propeptide, PINP; osteocalcin OC) und Resorptionsmarkern (C telopeptide, CTX; tartrate-resistant acid phosphatase 5, TRAP5b) nachgewiesen [25]. Der Zusammenhang zwischen dem low bone turnover und der gleichzeitig nachgewiesenen strukturellen Alteration (kortikale Porosität) ist zum gegenwärtigen Zeitpunkt unklar.

## Alternative (experimentelle) biochemische Marker der Knochenfragilität

Bei Diabetes ist in der Knochenmatrix der Gehalt von Pentosidin, dem am häufigsten vorhandenen AGE, im Vergleich zu nicht-diabetischen Menschen deutlich erhöht. Erhöhte Pentosidin-Spiegel im Knochen und im Serum korrelieren negativ mit der biomechanischen Stärke des Knochens (Abb. 2). Untersuchungen bestätigen erhöhte Werte von AGEs und auch von sRAGE (soluble receptors for advanced glycation endproducts) als prädiktiven Faktor für eine erhöhte Inzidenz von klinischen und vertebralen Frakturen unabhängig von der BMD [74].

Sclerostin, der endogene Inhibitor des Wnt/β-Catenin Signalweges und somit der Knochenformation durch Osteoblasten, ist bei T2DM deutlich erhöht (Abb. 2). Die Höhe der Sclerostin-Spiegel korreliert bei diesen Patient:innen mit der Inzidenz von Fragilitätsfrakturen [75]. Bei T1DM verhalten sich die Sclerostin-Spiegel genau gegenläufig zur Inzidenz von Frakturen. Patient:innen im obersten Drittel der gemessenen Spiegel hatten ein um 81 % geringeres Frakturrisiko verglichen mit Patient:innen mit Spiegeln im untersten Drittel [76]. Ob nun eine Erhöhung zirkulierender Sclerostin-Spiegel direkt die Dysfunktion von Osteozyten widerspiegelt und/oder ein Marker für eine zusätzliche Angiopathie sind, bleibt derzeit noch unbeantwortet [77].

Neben Sclerostin scheint auch ein weiterer Wnt-Inhibitor, Dickkopf‑1 (DKK-1) eine Rolle beim Knochenverlust bei Diabetes zu spielen (Abb. 2). Serumspiegel von DKK‑1 sind sowohl bei Kindern mit T1D also auch bei Erwachsenen mit T2DM erhöht [78–80]. Das Ausschalten von DKK‑1 in osteogenen Zellen in Mäusen führte zu einer Reduktion des durch T1DM-mediierten [81] Knochenverlusts.

Serum Periostin bzw. dessen Fragmente sind mit einem erhöhten Frakturrisiko bei nicht-diabetischen Patient:innen vergesellschaftet. Derzeit laufen Studien in großen diabetischen Populationen zur Evaluation dieses Markers [82]. Die Bestimmung von Serum exsosomalen microRNA (miRNA) Signaturen erscheint nicht nur bei diabetischen Populationen zukünftig eine entsprechende Option zu werden ([83, 84]; Abb. 2).

## Basisprophylaxe mit Vitamin D und Kalzium

Die Vitamin D- und Kalziumsubstitution ist sowohl eine eigenständige Therapiemöglichkeit der Osteoporose als auch die absolut notwendige Basis jeder spezifischen Osteoporosetherapie.

### Vitamin D

Eine ausreichende Versorgung mit Vitamin D ist eine wichtige Voraussetzung für die Knochengesundheit. Eine 25-OH-Vitamin D Serumkonzentration < 20 ng/ml (50 nmol/l) ist mit einem erhöhten Risiko für proximale Femurfrakturen und nichtvertebralen Frakturen verbunden [85].

Zur Therapie eingesetzt wird Cholecalciferol (Vitamin D3); 1 μg Vitamin D3 entspricht 40 IE Vitamin D3. Die Einnahme soll mit den Mahlzeiten erfolgen, da dies die Resorption verbessert.

Die Tagesdosis (z. B. 800 IE) kann auch als Wochenäquivalent gegeben werden (entsprechend zB. 5600 IE einmal wöchentlich). Im Einzelfall kann bei Malabsorption eine parenterale (intramuskuläre) Gabe von 100.000 IE Cholecalciferol notwendig sein. Die Gabe der aktiven Form von Vitamin D – Calcitriol (1,25-Dihydroxycholecalciferol) – ist nur bei schwerer Niereninsuffizienz indiziert.

Es gibt Hinweise auf eine positive Beeinflussung der diabetischen Insulinresistenz durch Vitamin D Supplementierung [86].

### Kalzium

Eine ausreichende Kalziumzufuhr, primär über die Nahrung, ist sicherzustellen.

Patient:innen mit Osteoporose (mit und ohne spezifischer Osteoporosetherapie) sollen daher täglich 1000 mg Kalzium aufnehmen, vorzugsweise über die Nahrung. Ist dies nicht möglich, sind Kalziumsupplemente erforderlich. Pro Einnahme wird eine Dosis von maximal 500 mg Kalziumsupplement empfohlen [61].

Calcium-Supplementierung könnte bei Menschen mit Diabetes möglicherweise zusätzliche positive Effekte bewirken, wie Linderung der Insulinresistenz, Verbesserung der Insulinsekretion, sowie Reduktion von Lipogenese und Entzündung [87].

## Spezifische Osteoporosetherapie bei Diabetes

Keine einzige randomisierte Studie hatte bisher als Endpunkt die Wirksamkeit einer spezifischen Osteoporosetherapie bei Patient:innen mit T2DM. Daher basieren die Empfehlungen für das Management von Patient:innen mit Diabetes und einem erhöhten Frakturrisiko auf empirischen Daten und der klinischen Erfahrung. Die klinische Evidenz in Bezug auf die Effizienz einer antiresorptiven oder anabolen Osteoporosetherapie bei gleichzeitigem Diabetes beruht daher auf post hoc Analysen von Subgruppen in großen randomisierten Osteoporose Studien und auch einer kleinen Anzahl von Observationsstudien [88].

Grundsätzlich sind sämtliche Medikamente zur Behandlung der Osteoporose auch bei Patient:innen mit einem manifesten Diabetes möglich und zugelassen. Da sowohl der Diabetes mellitus als auch die Osteoporose eine chronische Erkrankung mit einem dauerhaft erhöhten Risiko für sekundäre Komplikationen sind, ist die Indikation für eine langfristige Behandlung indiziert.

### Bisphosphonate

Bisphosphonate (Alendronat, Risedronat, Ibandronat, Zoledronat) sind potente Inhibitoren der Knochenresorption. Sie werden an metabolisch aktiven Umbaueinheiten im Knochen abgelagert und bewirken eine Apoptose von Osteoklasten. Die Resorptionsaktivität wird im Gesamtskelett deutlich gedämpft und das Frakturrisiko reduziert.

Oral werden Bisphosphonate nur in geringem Ausmaß (maximal 3 %) resorbiert; die Einnahme erfolgt stets nüchtern in ausreichendem Abstand zur Nahrungsaufnahme, mit ausreichend Wasser und in aufrechter Körperhaltung, um Irritationen der Ösophagusschleimhaut zu vermeiden.

Bei intravenöser Bisphosphonatgabe kann, überwiegend bei erstmaliger Verabreichung, eine sogenannte „Akutphasereaktion“ – im Wesentlichen ein grippeähnliches Zustandsbild mit Fieber und Muskelschmerzen – auftreten, die in der Regel innerhalb von 36 h nach intravenöser Gabe beginnt und dann 24–48 h anhält.

Bei allen Bisphosphonaten stellen die Hypokalzämie, eine erhebliche Nierenfunktionseinschränkung oder eine Gravidität eine Kontraindikation dar.

Bisphosphonate haben eine lange Verweildauer im Knochen. Residuale Wirkungen auf den Knochenstoffwechsel lassen sich auch nach Beendigung der Bisphosphonattherapie nachweisen. Das Auftreten von atypischen Femurfrakturen ist sehr selten, scheint aber unter einer Langzeitgabe mit Bisphosphonaten zuzunehmen. Kiefernekrosen sind bei dieser für Osteoporose zugelassenen Therapie eine mutmaßlich seltene Nebenwirkung. Eine Kontrolle des Zahnstatus ist allerdings vor Therapiebeginn empfehlenswert.

Es gibt keine durch Frakturdaten validierten individuellen Entscheidungskriterien für die Wiederaufnahme einer Therapie nach einer Therapiepause oder einen weiteren Therapieverzicht in Abhängigkeit von Veränderungen der BMD, der Knochenumbaumarker oder anderer messtechnischer oder klinischer Kriterien. Datenbankanalysen geben allerdings Hinweise auf einen Wiederanstieg des Knochenbruchrisikos nach Absetzen einer Bisphosphonattherapie [61].

### Denosumab

Denosumab ist ein monoklonaler Antikörper gegen RANKL, der die Reifung und Aktivierung der Osteoklasten hemmt. Es wird alle sechs Monate subkutan verabreicht und wird nicht renal eliminiert.

Bei der Behandlung der postmenopausalen Osteoporose ist eine Reduktion von vertebralen und nichtvertebralen Frakturen inklusive proximaler Femurfrakturen in Studien bis zu 10 Jahre nachgewiesen. Die Wirkung ist unabhängig von einer eventuellen Vorbehandlung mit Bisphosphonaten [89]. Die Behandlungsdauer ist unklar. Nach Absetzen von Denosumab scheint es im Gegensatz zu den Bisphosphonaten zu einem raschen Anstieg des Knochenumbaus und in weiterer Folge zu einer Abnahme der Knochenmineraldichte zu kommen. Kiefernekrosen und atypische Femurfrakturen sind bei dieser für Osteoporose zugelassenen Therapie eine mutmaßlich sehr seltene Nebenwirkung [61].

### Raloxifen

Raloxifen ist ein selektiver Östrogenrezeptor-Modulator (SERM), der die Knochenresorption hemmt und das Frakturrisiko für vertebrale Frakturen reduziert (nicht für nicht-vertebrale Frakturen und proximale Femurfrakturen). Raloxifen ist zugelassen für die Prävention und für die Therapie der Osteoporose bei postmenopausalen Frauen.

Ein bedeutender zusätzlicher Effekt ist die Reduktion des relativen Risikos eines invasiven (Östrogenrezeptor-positiven) Mammakarzinoms um 79 %. Eine unerwünschte Nebenwirkung ist die Erhöhung des thromboembolischen Risikos [61].

### Teriparatid

Teriparatid, ein aminoterminales Fragment des Parathormons, wird einmal täglich subkutan über 24 Monate angewandt. Der osteoanabole Effekt beruht auf einer Beschleunigung der Reifung und Stimulierung von Osteoblasten.

Im Anschluss an die anabole Reaktion des Knochens kommt es nach Beendigung der Teriparatid-Therapie wiederum zu einem gesteigerten Knochenabbau, weshalb eine sofortige Anschlussbehandlung mit einem Antiresorptivum (Bisphosphonat, Denosumab, SERM) unbedingt notwendig ist [61].

### Neue/zukünftige Osteoporose Medikamente

Romosozumab, ein Anti-Sclerostin Antikörper, verbessert die BMD und die Knochenstärke im diabetischen Rattenmodell. Studiendaten bei postmenopausalen Frauen mit einem erhöhten Knochenbruchrisiko zeigen eine außergewöhnlich starke Zunahme der BMD bei monatlicher Applikation. Daher könnte dieser Antikörper, der derzeit in Österreich in der Roten Box ist, zukünftig eine neue Behandlungsoption auch bei T2DM in der entsprechenden Indikation darstellen [90, 91]. Vor Beginn einer Therapie mit Romosozumab ist eine exakte Anamnese auf kardiovaskuläre Events zu erheben, da diese eine Kontraindikation darstellen.

## Management einer erhöhten Knochenfragilität bei Diabetes

Die Kriterien für den Beginn einer osteologischen Therapie bei Diabetes basieren entweder auf einer prävalenten Fragilitätsfraktur (unabhängig von der BMD) und/oder auf einer verminderten BMD. Das diagnostische Kriterium der Osteoporose in der DXA-Messung (T-score < 2,5) ist nicht mit der individuellen Therapieschwelle gleich zu setzen [61, 92].

Die wichtigste Entscheidungshilfe für den Beginn einer Therapie ist auch beim Patient:innen mit Diabetes eine prävalente Fragilitätsfraktur. Das Ziel ist jedoch, die Patient:innen vor der ersten niedrig-traumatischen Fraktur zu schützen.

### BMD Interventionsschwelle

**Figure 3:**
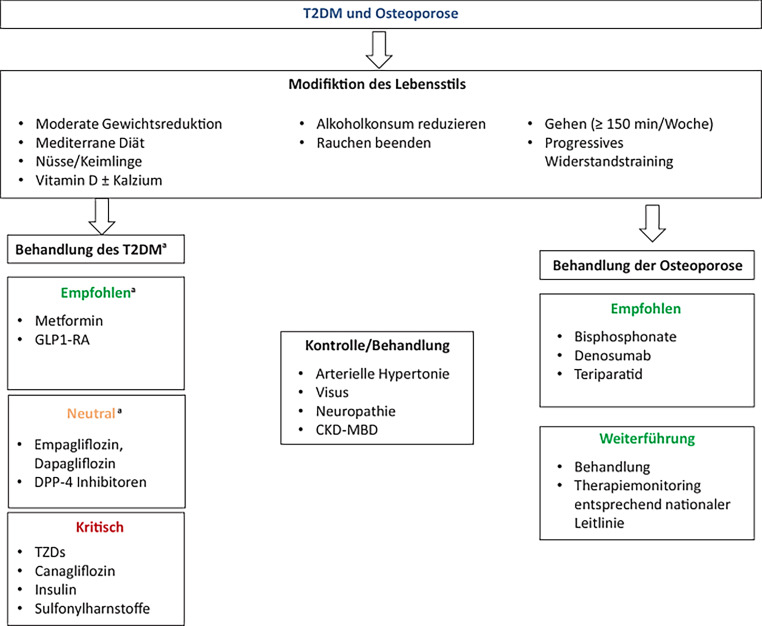

Bei Patient:innen mit einem manifesten T2DM unterschätzt die DXA-Messung das individuelle Frakturrisiko. Aktuell wird daher bei diesen Patient:innen eine Anhebung der Interventionsschwelle auf einen T‑score von −2,0 an der Lendenwirbelsäule (kumulativ L1–L4) oder an der Hüfte (Schenkelhals bzw. gesamte Hüfte) empfohlen, um der DXA-basierten Unterschätzung der Knochenfragilität entgegenzuwirken (Abb. 3). Diese Anhebung ist jedoch nur in westlichen Populationen zu empfehlen. Patient:innen aus Asien oder dem Nahen/Mittleren Osten haben bei Alters- und Geschlechts-adjustierter BMD niedrigere Frakturraten, die sich auch bei manifestem Diabetes auswirken.

Patient:innen mit einem ausgeprägten Verlust an BMD in zwei konsekutiven Messungen (= > 5 % in zwei Jahren) sollten schon bei Werten nahe der Interventionsschwelle prophylaktisch behandelt werden.

### FRAX®

FRAX, das WHO zertifizierte Fracture Risk Assessment Tool, implementiert nationale Frakturdaten und besteht aus 12 dichotomisierten Fragen (die 12. Frage zu BMD ist optional) [93]. Dem FRAX liegen klinische Risikofaktoren (clinical risk factor, CRF) zugrunde, die in randomisierten Studien ein erhöhtes individuelles Risiko für Fragilitätsfrakturen sind. FRAX berechnet zwei Werte: (a) eine 10-Jahreswahrscheinlichkeit für alle osteoporotischen Frakturen und (b) eine 10-Jahreswahrscheinlichkeit für eine osteoporotische Hüftfraktur. Entsprechend der Österreichischen Leitlinie zur Behandlung der Osteoporose wird ab einem Risiko von (a) ≥ 20 % bzw. (b) ≥ 5 % prophylaktisch eine knochenspezifische Therapie empfohlen.

Diabetes per se ist im FRAX kein eigener CRF, daher unterschätzt auch dieses diagnostische System die Frakturwahrscheinlichkeit bei einem manifesten Diabetes. Diabetes per se ist allerdings ein starker Risikofaktor für eine osteoporotische Fraktur, auch nach Korrektur aller CRFs und BMD [94]. FRAX bietet auch die Möglichkeit, die BMD-Werte mittels TBS-Korrektur zu rechnen. Vor allem bei T2DM führt dies zu einer Verbesserung der Vorhersagewahrscheinlichkeit.

Berechnungen mit dem FRAX Rechner haben gezeigt, dass es weitere Korrekturmöglichkeiten gibt, um sich bei T2DM unter Verwendung dieses Risikorechners dem individuellen Frakturrisiko zu nähern. Eine Möglichkeit ist (a) die Erhöhung des Patient:innenalters um 10 Jahre, da der Risikofaktor Diabetes bei dieser Korrektur in etwa dem Risikofaktor Alter (als etablierter CRF im FRAX) entspricht. Die andere Möglichkeit ist (b) die Verminderung des gemessenen T‑cores am Schenkelhals um 0,5 Standardabweichungen zu verringern (z. B. T‑core −2,4 statt den tatsächlich gemessenen −1,9). Eine weitere Möglichkeit wäre es, (c) Rheumatoide Arthritis als CRF zu nehmen. Der Untersucher sollte sich für eine der drei Möglichkeiten entscheiden, jedoch nicht alle drei Optionen verwenden.

Nach aktueller Datenlage bieten diese drei Optionen trotz aller methodischen Limitationen derzeit die beste Lösung, sich dem tatsächlichen individuellen Frakturrisiko bei T2DM zu nähern. Die größte Trefferwahrscheinlichkeit bietet die Verwendung des CRF Rheumatoide Arthritis und wird daher derzeit auch von der Task Force Diabetes der International Osteoporosis Foundation empfohlen ([88]; Tab. 3).

## Stellenwert der Physikalischen Medizin und Rehabilitation bei Prophylaxe und Therapie

### Rehabilitative Problemstellung

Diabetes birgt neben der erhöhten Gefahr für Frakturen ein Risiko für zahlreiche weitere Folgeerscheinungen, wie koronare Herzerkrankung, Schlaganfall, periphere vaskuläre Komplikationen, Neuropathie, oder Retinopathie [95, 96]. Diese Folgeerscheinungen erhöhen nicht nur die Mortalität, sondern bewirken auch Dekonditionierung, erhöhtes Sturzrisiko und zunehmenden Verlust der Fähigkeit, Aktivitäten des täglichen Lebens (ATLs) selbstständig durchzuführen. Deshalb ist es wichtig, die Krankheit möglichst frühzeitig rehabilitativ zu beeinflussen.

Körperliche Aktivität resultiert generell in einer gesundheitswirksamen Verbesserung der körperlichen Leistungsfähigkeit, besonders profitieren jedoch Personen mit metabolischem Syndrom, da die positive Beeinflussung der Insulinresistenz einen zentralen Wirkungsmechanismus der Trainingstherapie darstellt [97].

Es gilt bei der Planung und Durchführung der Bewegungstherapie einige potenzielle Folgen des Diabetes mellitus zu berücksichtigen, wie Retinopathie, Neuropathie, kardiale Erkrankungen oder Hypoglykämien [97].

In Tab. 4 findet sich eine „best practice“ Empfehlung der Autoren dieser Leitlinie, die sich sowohl für Prophylaxe als auch Therapie eignen.

**Table 4:** 

Trainingsart	Umsetzung	Beispiele
Krafttraining großer Muskelgruppen	2–3 × wöchentlich; 8 Wiederholungen; Widerstand, mit dem 10 Wiederholungen durchgeführt werden können; 2 Sets; 1 min Pause	Kniestrecker, Kniebeuger Gesäß‑, Bauch‑, Rücken‑, Schulterblatt‑, Arm- und Nackenmuskulatur
Ausdauertraining	4 × wöchentlich 40 min; Intensität, bei der kurzes Sprechen noch möglich ist	Flottes Gehen, Nordic Walking, Schwimmen, Radfahren, Skilanglauf, lockeres Wandern
Beweglichkeitsübungen	3–7 × wöchentlich	– täglich: Radfahren im Liegen, Vorfüße kreisen, Arme kreisen, Schultern kreisen, Kopf drehen und Nicken – 3 ×/Woche Dehnen, 2 × 30–60 sec halten dazwischen locker lassen: Waden, Hüftstrecker, Hüftbeuger, Adduktoren, Rumpf, Pectoralis, Nacken – 3 ×/Woche Mobilisieren: „Katzenbuckel“ für LWS/BWS, Rotation plus Nicken sowie Seitneigen für HWS, Arme im Liegen über Kopf ablegen für Schultern, aufgestellte Beine im Liegen nach re/li ablegen, Kopf dreht in die Gegenrichtung, jeweils 5 Wiederholungen
Koordinationstraining/Sturzprophylaxe (insbesondere bei Sturzrisiko)	3 × wöchentlich	Einbeinstand mit/ohne Anhalten, auf einem Strich gehen, rückwärtsgehen, Stehübungen auf labilen Unterlagen, zB Therapiekreisel, Gehen auf Zehenspitzen, Gehen auf Fersen, 30 s bis 2 min pro Übung, 2–5 Wiederholungen

Die Tabelle enthält Trainingsempfehlungen für Prophylaxe und Therapie. Diese Empfehlungen sind als „best practice“ Vorschläge der Autoren dieser Leitlinie zu sehen. Die Adaptierung (Widerstand) definiert sich durch die gewählte Belastung in Kilogramm. Die Frequenz (Einheiten pro Woche, Wiederholungsanzahl, etc.) ist davon unbeeinflusst. Das Übungsniveau (z. B. offene oder geschlossene Augen bei Balancetraining) ist sowohl im präventiven als auch im rehabilitativen Setting individuell anzupassen. Anzumerken ist, dass während des Trainings ein erhöhtes Sturzrisiko besteht und deshalb Sicherheitsvorkehrungen getroffen werden sollten. Insbesondere kann ein zu hohes Übungsniveau zu Beginn (z. B. Balancetraining mit geschlossenen Augen oder dual-task Übungen) zu fordernd sein kann und somit Stürze während des Trainings begünstigen

Die folgenden Referenzen verweisen auf trainingstherapeutische Empfehlungen weiterer nationaler und internationaler Leitlinien: [85, 97–99].

### Krafttraining

Durch regelmäßiges Krafttraining kommt es über einen zusätzlichen Glucosetransporter zu einer Steigerung der zellulären Glucoseaufnahme, was in einer Erhöhung des Grundumsatzes und somit günstigen Gewichtsentwicklung resultiert. Dies ist insbesondere bei sarkopenen adipösen („sarcopenic obesity“) Patient:innen von Bedeutung [97].

Krafttraining steigert die Muskelmasse und wirkt osteoanabol [100, 101]. Sollte aufgrund kardiorespiratorischer Einschränkungen nur ein geringer Trainingsumfang möglich sein, gilt Krafttraining im Vergleich zu Ausdauertraining als leichter einsetzbar [97]. Zusätzlich scheint sich Krafttraining positiv auf das Sturzrisiko auszuwirken [102].

In einem diabetischen Rattenmodell mit via Elektrostimulation simuliertem Krafttraining zeigten sich Verbesserungen von Knochendichte und Mikroarchitektur [103]. Bei diabetischen Kindern kam es nach einem 9‑monatigen Training (2 ×/Woche 90 min, Ballsport, springen, Gymnastik) zu einer Verbesserung der Ganzkörper- und lumbalen Knochendichte [104]. Bei älteren Personen mit Typ 2 Diabetes wurden nach 12-monatigem Gewichtsreduktionsprogramm kombiniert mit progressivem Krafttraining (3 ×/Woche, 3 Sätze, 8–10 Wiederholungen mit 75–85 % des 1‑Wiederholungsmaximums; erste 6 Monate supervidiert mit freien Gewichten und Geräten, zweite 6 Monate heimbasiert mit Kurzhanteln und Gewichtsmanschetten) eine Stabilisierung der Knochendichte beschrieben, verglichen mit reiner Gewichtsreduktion [105]. Bei postmenopausalen Frauen mit Prädiabetes und Typ 2 Diabetes kam es durch ein 32-wöchiges Training (Gehen, Wassergymnastik und Krafttraining: Kurzhanteln, Gewichtsmanschetten, 6 Übungen, 3 Sätze, 15–20 Wiederholungen) zu einer Verbesserung der Knochendichte am Wardschen Dreieck [106]. Eine aktuelle Studie bei Personen mit Typ 2 Diabetes fand nach einem 12-monatigem Trainingsprogramm (5–6 ×/w aerobes Training, sowie 2–3 ×/w 30 min Krafttraining ohne detailliertes Schema) von einer Stabilisierung der Knochendichte [107].

### Ausdauertraining

Ausdauertraining bewirkt eine Effizienzsteigerung der Aufnahme und Aufnahme und des Metabolismus von Glukose in der Muskulatur. Dies resultiert in einer Verbesserung der Insulinresistenz [97].

Weiters könnte aerobes Ausdauertraining eine positive Wirkung auf Knochenstoffwechselparameter haben [108, 109]. Zudem könnte es den altersbedingten Verlust an Knochenmasse verlangsamen, eine eindeutige positive Wirkung auf die Knochendichte wurde jedoch nicht berichtet [110, 111]. Jedenfalls scheint regelmäßiges aerobes Training, welches in höherer aerober Fitness resultiert, die Erholung nach intensiven Aktivitäten zu beschleunigen [112].

### Balancetraining

Balancetraining ist eine wirksame Strategie, um das Risiko für Stürze zu reduzieren [113]. Dies ist insbesondere bei Menschen mit Diabetes von großer Bedeutung, da in dieser Personengruppe nicht nur ein erhöhtes Sturzrisiko, sondern auch die Angst vor Stürzen weit verbreitet sind [114, 115]. Eine rezente Arbeit konnte zeigen, dass ein 3‑monatiges Balancetraining eine deutliche Verbesserung zahlreicher Parameter der Stabilität sowie des Sturzrisikos bewirkt [115].

### Beweglichkeitstraining

Eine Verbesserung der Beweglichkeit geht mit einem geringeren Risiko für Stürze einher [116].

### Weitere Maßnahmen

Ergotherapeutische Interventionen können dabei helfen, nicht nur die glykämische Kontrolle, sondern auch die psychosoziale Situation zu verbessern. Insbesondere konnten Verbesserungen des HbA1c und der Diabetes-bezogenen Lebensqualität gezeigt werden [117].

### Rehabilitation nach Frakturen

In der Rehabilitation von Personen mit osteoporotischen Frakturen ist es wichtig, die orthopädisch-traumatologischen Aspekte mit den oben erwähnten Maßnahmen zu kombinieren. Menschen mit Diabetes weisen hierbei eine hohe Vulnerabilität auf. Dies spiegelt sich nach operativer Versorgung von Schenkelhalsfrakturen in einem häufigeren Transfer an ICU-Einheiten, häufigerer Wiederaufnahme im Spital nach erfolgter Entlassung sowie einer höheren ein-Jahres-Mortalität wider [118].

### Wirbelkörperfraktur

Bei osteoporotischen Wirbelkörperfrakturen sollten eine multimodale Schmerztherapie (medikamentös und physikalisch), bei unzureichender Stabilität eine Miederversorgung (meist für 8–12 Wochen) sowie ein rasches Wiedererlangen der Mobilität erfolgen, um Komplikationen der Immobilität, wie Pneumonie oder Dekonditionierung, zu verhindern [119].

In der Frühphase erfolgen im Rahmen der Bewegungstherapie neben der Remobilisierung auch Instruktionen bzgl. Ergonomie (zB. en bloc Drehen), isometrische Stabilisierungsübungen zur Verhinderung einer Kyphosierung, sowie ggf. ein stufenweiser Abbau des Mieders [120].

Weiters sollte ein Training der Balance erfolgen, um das Risiko für weitere Stürze zu reduzieren, sowie die Beseitigung von Stolperfallen und das Absetzen von Sturz-begünstigenden Medikamenten [120].

Funktionelle Orthesen können Schmerzen lindern, die Rückenmuskulatur aktivieren und die Aktivitäten des täglichen Lebens (activities of daily living, ADL) erleichtern [119].

Bei osteoporotischen Wirbelkörperfrakturen kommt es im Allgemeinen gemäß radiologischer Kriterien nach etwa drei bis vier Monaten zur Ausheilung [121]. Bei Diabetes mellitus besteht jedoch eine verzögerte Frakturheilung [122]. Jedenfalls soll ab der radiologisch bestätigten Heilung auch an der Beweglichkeit der Wirbelsäule gearbeitet werden.

Kommt es aufgrund der Fraktur zu funktionellen Einschränkungen, sollten ergotherapeutische Interventionen bzgl. ADL und Hilfsmittel erfolgen, um die Selbstständigkeit wiederzuerlangen. Bei schwerwiegenden Einschränkungen ist ggf. eine pflegerische Unterstützung und/oder psychosoziale Intervention erforderlich [120].

Nach osteoporotischen Wirbelkörperfrakturen kann eine individualisierte und supervidierte Trainingstherapie vorsichtig ab etwa 4–6 Wochen (in Abhängigkeit der klinischen Beschwerden und dem radiologischen Heilungsverlauf) begonnen werden [123, 124]. Hierbei soll in der Akutphase (erste 3 Monate) der Fokus auf die Aktivierung und Verbesserung der Ausdauer der Rückenstrecker gelegt werden, sowie auf ein Balancetraining. Zusätzlich können beispielsweise die Kniestrecker und/oder Kniebeuger ohne fortgeleitete Belastung der Wirbelsäule (z. B. mittels leg extension bzw. leg curl) trainiert werden. Die Deutsche Gesellschaft für Orthopädie und Unfallchirurgie (DGOU) empfiehlt bereits ab 6 Wochen mit einem intensivierten Muskelaufbautraining mit Geräten zu beginnen [124]. Jedenfalls soll in der subakuten Phase (ab ca. 3 Monaten, bzw. ab erfolgter Frakturheilung) das Balancetraining intensiviert und um funktionelles Training bzw. Krafttraining ergänzt werden [123].

Supervidiertes Krafttraining bei postmenopausalen Frauen [125] und Männern [126] mit sehr niedriger Knochendichte scheint sicher zu sein. Insbesondere wurde in den LIFTMOR-Studien von einer Verbesserung von Kyphosen berichtet, es traten keine neuen Frakturen auf und es kam auch zu keinem Progress prävalenter Frakturen.

### Schenkelhalsfraktur

Die Schenkelhalsfraktur ist meist ein Zeichen für komplexe Funktionsstörungen. Deshalb sind die rehabilitativen Ziele vielfältig und es sollten medikamentöse, diätetische, bewegungs- und ergotherapeutische Maßnahmen sowie physikalische Modalitäten kombiniert werden [127, 128].

Eine Atemtherapie zur Pneumonieprophylaxe sollte frühzeitig zum Einsatz kommen, unserer Meinung nach bereits präoperativ. Die Remobilisierung soll rasch erfolgen, bereits ab dem ersten postoperativen Tag und mindestens einmal täglich [129].

Die Belastbarkeit sowie die freigegebenen Bewegungsumfänge variieren abhängig von der Operationsmethode. Die Bewegungstherapie gestaltet sich außerdem entsprechend den Phasen der Bindegewebsheilung [128, 130]: In der Akutphase (Tage 0–5) stehen Atemtherapie, entstauende Maßnahmen, passiv/assistive Mobilisation und Stehversuche im Vordergrund, wohingegen in der Proliferationsphase (Tage 5–21) an der weiteren Mobilisierung, Koordinations- und Sensomotoriktraining, Verbesserung der Beweglichkeit, und des Ganges gearbeitet wird. In der Konsolidierungsphase (Tage 21–60) erfolgen v. a. Narbentherapie, Kräftigung und Sturzprophylaxe. In der Umbauphase liegt der Schwerpunkt auf der Medizinischen Trainingstherapie.

## Footnotes

1 Aus Gründen der besseren Lesbarkeit wird bei Personenbezeichnungen und personenbezogenen Hauptwörtern auf dieser Website die männliche Form verwendet. Entsprechende Begriffe gelten im Sinne der Gleichbehandlung grundsätzlich für alle Geschlechter.

## Interessenkonflikt

C. Muschitz hat von folgenden Firmen, die auch fördernde Mitglieder der ÖGKM (Österr. Gesellschaft für Knochen- und Mineralstoffwechsel) und der ÖDG sind, Honorare für Vorträge und Beratung sowie wissenschaftliche Grants erhalten: Amgen Österreich; Eli Lilly Österreich, Novartis Pharma Österreich. Es besteht kein Konflikt mit der Erstellung dieses Manuskripts. A. Kautzky-Willer hat von folgenden Firmen, die teils auch fördernde Mitglieder der ÖDG sind, Honorare für Vorträge und Beratung erhalten: AstraZeneca, Amgen, Boehringer Ingelheim, Eli Lilly, Merck Sharp & Dohme, Novartis, Novo Nordisk, Roche, sanofi-aventis, Servier, Takeda. Es besteht kein Konflikt mit der Erstellung dieses Manuskripts. Y. Winhofer hat von folgenden Firmen, die auch teils auch fördernde Mitglieder der ÖDG sind, Honorare für Vorträge und Beratung erhalten: Amgen, Astra Zeneca, Eli Lilly, Novartis, Sanofi. Es besteht kein Konflikt mit der Erstellung dieses Manuskripts. M. Rauner hat von folgender Firma, die auch fördernde Mitglieder der ÖGKM ist, Honorare für Vorträge und Beratung erhalten: Amgen. Es besteht kein Konflikt mit der Erstellung dieses Manuskripts. J. Haschka hat keine Honorare von Firmen für Vorträge oder Beratung erhalten. D. Cejka hat von folgenden Firmen, die auch fördernde Mitglieder der ÖDG sind, Honorare und/oder Grants erhalten: Amgen, UCB, Stada. Es besteht kein Konflikt mit der Erstellung dieses Manuskripts. Advisory Board for Amgen, Gedeon Richter, Stada and UCB. Speaker for UCB and Takeda. Research grant from Amgen. R. Wakolbinger-Habel hat von folgenden Firmen, die auch fördernde Mitglieder der ÖGKM sind, Honorare für Vorträge erhalten: Amgen, UCB. Es besteht kein Konflikt mit der Erstellung dieses Manuskripts. P. Pietschmann hat von folgenden Firmen, die teilweise auch fördernde Mitglieder der ÖGKM sind, Honorare und/oder Forschungsförderungen erhalten: Amgen GmbH, Biomedica GmbH, Fresenius Kabi Austria, Takeda Pharma GesmbH, TAmiRNA GmbH, UCB Biopharma Srl/UCB Pharma. Es besteht kein Konflikt mit der Erstellung dieses Manuskripts.
